# Circulating microRNAs and DNA Methylation as Regulators of Direct Oral Anticoagulant Response in Atrial Fibrillation and Key Elements for the Identification of the Mechanism of Action (miR-CRAFT): Study Design and Patient Enrolment

**DOI:** 10.3390/jpm14060562

**Published:** 2024-05-24

**Authors:** Georgia Ragia, Thomas Thomopoulos, Georgios Chalikias, Athanasios Trikas, Dimitrios N. Tziakas, Vangelis G. Manolopoulos

**Affiliations:** 1 Laboratory of Pharmacology, Medical School, Democritus University of Thrace, Dragana Campus, 68100 Alexandroupolis, Greece; gragia@med.duth.gr; 2 Individualised Medicine & Pharmacological Research Solutions (IMPReS) Center, Dragana Campus, 68100 Alexandroupolis, Greece; 3 Department of Cardiology, “Elpis” General Hospital of Athens, 11522 Athens, Greece; thomopoulosthomas86@gmail.com (T.T.); atrikas@otenet.gr (A.T.); 4 Cardiology Department, Medical School, Democritus University of Thrace, Dragana Campus, 68100 Alexandroupolis, Greece; gchaliki@med.duth.gr (G.C.); dtziakas@med.duth.gr (D.N.T.); 5 Clinical Pharmacology Unit, Academic General Hospital of Alexandroupolis, Dragana Campus, 68100 Alexandroupolis, Greece

**Keywords:** direct oral anticoagulants (DOACs), DNA methylation, microRNAs, epigenetics, miR-CRAFT, coagulation, pathways, pleiotropic effects

## Abstract

Direct oral anticoagulants (DOACs) are the standard treatment for thromboembolic protection in atrial fibrillation (AF) patients. Epigenetic modifications, such as DNA methylation and microRNAs, have emerged as potential biomarkers of AF. The epigenetics of DOACs is still an understudied field. It is largely unknown whether epigenetic modifications interfere with DOAC response or whether DOAC treatment induces epigenetic modifications. To fill this gap, we started the miR-CRAFT (Circulating microRNAs and DNA methylation as regulators of Direct Oral Anticoagulant Response in Atrial Fibrillation) research study. In miR-CRAFT, we follow, over time, changes in DNA methylation and microRNAs expression in naïve AF patients starting DOAC treatment. The ultimate goal of miR-CRAFT is to identify the molecular pathways epigenetically affected by DOACs, beyond the coagulation cascade, that are potentially mediating DOAC pleiotropic actions and to propose specific microRNAs as novel circulating biomarkers for DOAC therapy monitoring. We herein describe the study design and briefly present the progress in participant enrolment.

## 1. Introduction

Atrial fibrillation (AF) is the most common arrhythmia and a major factor in stroke and stroke-associated mortality, affecting more than 37 million people worldwide. In Greece, it is estimated that more than 150,000 patients present with non-valvular AF, accounting for 3% of the total adult population [1]. The oral anticoagulants indicated for AF include the vitamin K antagonists warfarin, acenocoumarol and phenprocoumon, and direct oral anticoagulants (DOACs). Since their introduction into clinical practice, DOACs have dominated over the older vitamin K antagonists and are currently the standard treatment for non-valvular AF and venous thromboembolism (VTE). The approved DOACs in Greece are the direct thrombin inhibitor, dabigatran, and the factor Xa inhibitors rivaroxaban, apixaban, and, more recently edoxaban. 

DOACs intervene directly in the coagulation cascade. Dabigatran targets thrombin, whereas factor Xa inhibitors inhibit the prothrombinase complex-bound and clot-associated factor X, resulting in thrombin reduction. Thus, the anticoagulant effect of DOACs is exerted via the inhibition of blood clotting. Since thrombin is well known to activate protease-activated receptor (PAR) signaling, DOACs potentially exert effects beyond anticoagulation [2]. 

For vitamin K antagonists, it is well known that a great variation in response and required drug dose exists among individuals and that this interindividual dose variability is influenced significantly by genetic variations [3,4]. The application of vitamin K antagonist pharmacogenomics has two dimensions: both predicting the optimal dosing of these drugs and additionally identifying individuals who are at increased risk of bleeding, and, consequently, would benefit from a DOAC rather than vitamin K antagonists [5]. 

DOACs differ from each other in their pharmacology and pharmacokinetics. The variation in bleeding and thrombotic events noted with DOACs under fixed-dose conditions also suggests inter-individual pharmacodynamic differences. In addition to other factors, the presence of common genetic variants and drug–drug interactions may contribute to these differences. Several studies on DOAC pharmacogenomics have been published to date. Two genome-wide association studies on dabigatran have shown that CES1 rs2244613 minor allele was associated with lower exposure to active dabigatran metabolite and with a lower risk of bleeding [6], and that, in Chinese patients, UBASH3B rs2276408 and FBN2 rs3805625 were related to bleeding, and 17 SNPs were related to dabigatran pharmacodynamics [7]. Other studies have shown that CES1 rs8192935 or rs71647871 and polymorphisms in 16 genes (SLC4A4, FRAS1, SULT1A1, ABCB1, ABCC2, ABCG2, CYP2B6, CYP1A2, CYP2C19, CYP3A5, CES1, SLCO1B1, SLC22A1, UGT1A1, UGT1A9, and UGT2B7) were significantly associated with dabigatran plasma concentrations and metabolism [8,9,10,11]. For rivaroxaban and apixaban, ABCB1 polymorphisms were associated with thromboembolic and bleeding risk [12,13], whereas ABCG2 polymorphisms were associated with apixaban pharmacokinetics [14,15]. Despite these findings, current evidence does not support DOAC pharmacogenomics implementation.

Beyond the effect of genetic variations on drug response, the dynamic state of gene expression can also interfere with inter-individual responses to pharmacotherapy. Gene expression is modified by non-permanent alterations to the DNA and other mechanisms modulating the fate of gene transcripts, referred to as epigenetic modifications [16]. Among epigenetic modifications, microRNAs and DNA methylation regulate gene expression and can also affect or can be affected by drug treatment [16]. MicroRNAs (miRs) are a highly conserved group of small, non-coding RNA molecules that have been found to control gene expression. In AF, miRs have drawn great attention as potential noninvasive biomarkers of disease. Evidence has been emerging that differences in circulating miRs expression are associated with AF development and progression [17,18,19,20]. In anticoagulation, the role of miRs has not yet been elucidated. Evidence shows that fibrinogen, tissue factor, and antithrombin are regulated by miRs [21]. DNA methylation consists of the addition of a methyl group in cytosines located in CpG islands of the promoter regions. Heavily methylated promoters suppress gene expression, whereas demethylated promoters favor the binding of transcription factors, thus enabling gene expression [22]. Studies focus on building a DNA methylation biomarker-based prediction model for AF based on tissue-specific gene methylation [23]. Blood DNA methylation levels have been linked to circulating fibrinogen levels, supporting the idea that epigenetic studies can help in identifying pathways shared by coagulation factors, anticoagulation, and disease pathogenesis [24]. 

The expression profile of miRs and DNA methylation in AF patients treated with DOACs has been scarcely studied. It was just recently found that miR-320a and miR-483 levels are associated with the pharmacokinetic and pharmacodynamic profiles of rivaroxaban [25], whereas no studies are currently available on the potential association between DOAC therapy and alterations in DNA methylation levels. It appears, thus, that DOAC epigenomics is a naïve, albeit crucial in mechanism identification, field of research. 

To fill this gap, we initiated the miR-CRAFT (Circulating microRNAs and DNA methylation as regulators of Direct Oral Anticoagulant Response in Atrial Fibrillation) research study. miR-CRAFT investigates the dynamic expression of miRs and alterations in DNA methylation during DOAC therapy.

The specific aims of this miR-CRAFT study are as follows:

1. To study the differential expression of miRs and DNA methylation in naïve AF patients starting DOAC therapy, comparing between and within patients and between and within each DOAC drug received.
2. To construct and explore the DOAC regulatory network of miRNA-mRNA-genes.
3. To compare the baseline expression of miRs and DNA methylation between AF patients and controls.
4. To identify novel biomarkers that can be further studied for their association with DOAC response.

The ultimate goal is to identify the molecular pathways epigenetically affected by DOACs beyond the coagulation cascade that potentially mediate DOAC pleiotropic actions and propose specific miRs as novel circulating biomarkers for DOAC therapy monitoring.

## 2. Materials and Methods

### 2.1. Hospital Clinics Information and Protocol Approval

Participants are enrolled in the cardiology department of Athens General Hospital “Elpis” (approval ΕΣ 23/14 April 2019) and of the Academic General Hospital of Alexandroupolis (approval ΕΣ 3/3 February 2022) in Greece. Both hospitals are well known for their well-established Cardiology Department. The cardiologists involved in this miR-CRAFT study have extensive experience in AF diagnosis and treatment, and have participated in several clinical trials, ensuring good clinical practice and smooth work-flow regarding patient enrolment, treatment, and monitoring. All participants provided written informed consent to participate in the study, to provide a small volume of whole blood for DNA extraction and miRNA purification and analysis, and to allow anonymized access to their medical history. The study is being conducted according to the Declaration of Helsinki. 

### 2.2. Design of the Study

This is a prospective cohort study that does not include an intervention arm. All participants will be treated according to standard treatment. Patients and controls will be examined by an experienced cardiologist who is responsible for AF diagnosis, DOAC choice, and patient follow-up and monitoring. 

### 2.3. Study Population

**Figure 1:**
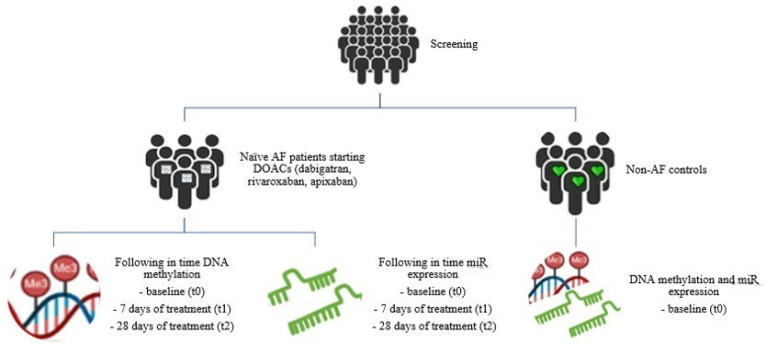
miR-CRAFT study design. After initial screening, newly diagnosed atrial fibrillation patients eligible for direct oral anticoagulant (DOAC) treatment and non-AF individuals will be enrolled in the study. In patients, DNA methylation and microRNAs expression will be followed over time to identify changes from baseline to 7 days and 28 days of DOAC therapy. Baseline comparisons of DNA methylation and microRNAs expression between patients and controls will be made.

In this miR-CRAFT study, naïve non-valvular AF patients eligible for DOAC treatment are prospectively enrolled in two cardiology departments, as described earlier. Patients treated with dabigatran or rivaroxaban or apixaban will be included in the study. Candidate participants that fulfill the inclusion criteria will be informed on site of the aims of the study by the cardiologists of each hospital upon admission and at the time of diagnosis. Inclusion criteria are newly diagnosed adult patients (age ≥ 18 years) with non-valvular AF (long-standing, persistent, or paroxysmal) and no previous use of anticoagulant drugs. Diagnosis will be confirmed with 12-lead electrocardiography (ECG) [26]. The reference group consists of sex- and age-matched individuals presenting for an evaluation of chest discomfort or elective cardiac surgery, who have a normal sinus rhythm, have no evidence of any acute coronary event or heart failure, and have no history of AF. Insulin treatment and cancer are exclusion criteria for both patients and controls, as they both interfere with epigenetic alterations. The follow-up period will consist of 28 days and a total of three visits; the first visit will be upon admission to the hospital and AF diagnosis (t0, baseline), the second visit will be on day 7 of DOAC treatment, and the third visit will be on day 28 of DOAC treatment (t2). An outline of this miR-CRAFT study is presented in Figure 1.

### 2.4. Sample Size Calculation

In miRs expression analysis, a priori power calculation is difficult to perform; however, to detect a 1.5-fold change in expression, a sample size of 19 subjects per group is suitable for achieving an 80% average power with a false discovery rate of 10% and an estimated proportion of non-differentially expressed miRs of 0.83 [27]. Assuming a 10% dropout rate, we will include at least 66 AF patients, 22 patients in each of the dabigatran, rivaroxaban, and apixaban treatment groups. 

### 2.5. Data Collection

For all participants, demographic (age, gender, height, weight, smoking habits), biochemical (urea, creatinine, SGOT, SGPT, hemoglobin, platelets count), and clinical data (CHA_2_DS_2_-VASc score, type of AF) will be collected, as well as personal medical history including co-morbidities (hypertension, diabetes, dyslipidemia) and co-medications. Non-adherence to DOAC treatment is expected by the attending cardiologist via interview during the examination. Adverse events, including thromboembolic and bleeding events, will be recorded [28,29]. Since miR-CRAFT aims to identify mechanistic pathways, minor bleedings will also be recorded, albeit they are clinically insignificant.

### 2.6. Sampling

From all participants, 2 blood samples (approximately 3 mL) will be collected by direct venipuncture from each patient into a vacutainer tube containing ethylenediaminetetraacetic acid (EDTA) at 3 different time-points: at the time of diagnosis (t0), on the 7th day of anticoagulation (t1), and on the 28th day of treatment (t2). Blood samples on t1 and t2 will be drawn in the morning and prior to the scheduled drug dosage (trough levels).

### 2.7. Laboratory Procedures

Fresh blood sample will be centrifuged for 10 min at 2000× *g*; plasma will be removed without disturbing sedimented cells and stored at −80 °C upon microRNA isolation. NucleoSpin^®^ miRNA Plasma (Macherey-Nagel, Düren, Germany) will be used for plasma miR purification. TaqMan™ Advanced miRNA cDNA Synthesis Kit (ThermoScientific, Waltham, MA, USA) will be used for cDNA synthesis and miR pre-amplification. Genomic DNA will be extracted from fresh whole blood by using the FDA-approved MagCore^®^ Genomic DNA Whole Blood Kit in a MagCore Automated Nucleic Acid Extractor (RBC Bioscience, New Taipei City, Taiwan) within 72 h of blood collection and will be stored at −20 °C until use. 

### 2.8. Phase I: Untargeted Analysis

In phase I untargeted analysis, only male participants will be included to reduce the gender confounders that potentially affect miR expression. For 12 male participants (4 treated with dabigatran, rivaroxaban, or apixaban) matched for age and co-morbidities, miRNome analysis will be conducted by using TaqMan™ OpenArray™ Human Advanced MicroRNA Panel (ThermoFisher Scientific, Waltham, MA, USA), a high-throughput approach that allows for the simultaneous analysis of 754 well-characterized human miR sequences. Analysis will be conducted on QuantStudio™ 12K Flex Real-Time PCR System (ThermoFisher Scientific). MiRs that show significant differences in their expression among study time-points, after Benjamini–Hochberg false discovery rate adjustments, will be further assessed in Phase II analysis.

### 2.9. Phase II: Targeted Analysis

Phase II analysis will include validating phase I miR results in total patient cohort, as well as targeted, hypothesis-driven miR and DNA methylation analysis. In Phase II analysis, the results generated by Phase I, as well as additional miRs that will be chosen based on biological hypotheses or from evidence derived from published studies, will be assessed in the total population cohort by using predesigned hsa-miR TaqMan assays (ThermoFisher Scientific). As endogenous controls, miRs constitutively expressed in plasma will be used. Additionally, in Phase II, the targeted DNA methylation analysis of gene promoters will be assessed by using SYBR Green-based quantitative methylation-specific polymerase chain reaction (PCR) (qMSP) after bisulfite conversion of genomic DNA. The selection of genes for targeted methylation analysis will be guided by the biological pathways revealed in the Phase I results. Additionally, hypothesis-driven genes playing crucial roles in pathways involved in DOAC pleiotropy, such as inflammation and endothelial integrity, will be included in DNA methylation analysis. 

### 2.10. Statistical Analysis

The relative miR expression will be quantified using the comparative Ct (ΔΔCt) method between or within patients at the same or at different time-points. Analyses will be carried out on the total patient cohort, as well as after patients are stratified according to DOAC treatment (dabigatran vs. rivaroxaban vs. apixaban, thrombin inhibitor vs. factor Xa inhibitors), gender, and incidence of AEs. Dichotomous variables will be created (up-/down-regulation of expression/methylation) and the appropriate unpaired and paired *t*-tests will be used for comparing continuous variables. The unpaired two-tailed *t*-test or the one-way ANOVA test will be used to compare groups with continuous data, as appropriate. Where applicable, regression logistic analysis, before and after adjustment for other factors (e.g., demographics, gender, co-morbidities and co-medications) known to affect the studied condition (e.g., adverse event, miR up- or down-regulation, DNA demethylation), will be used to calculate the odds ratio. A *p*-value of less than 0.05 will be used for significance and raw *p*-values will be adjusted using the Benjamini–Hochberg method. Fold changes within groups will be calculated. Post hoc power calculation will be performed. 

### 2.11. Bioinformatics Analysis

Genes targeted by the differentially expressed miRs will be screened out by using publicly available miR databases. The predicted target genes and proteins will be further analyzed by pathway enrichment analysis and a regulatory network of differentially expressed miRs and their target genes will be constructed by using the NetworkAnalyst tool. For the detection of physical and functional interactions of genes, protein–protein interaction networks will be created using two different online tools: Search Tool for the Retrieval of Interacting Genes/Proteins (STRING) and NetworkAnalyst. Visualizations via HeatMaps and networks will be performed with the appropriate applications. 

## 3. Results

### 3.1. Patient Enrolment

**Figure 2:**
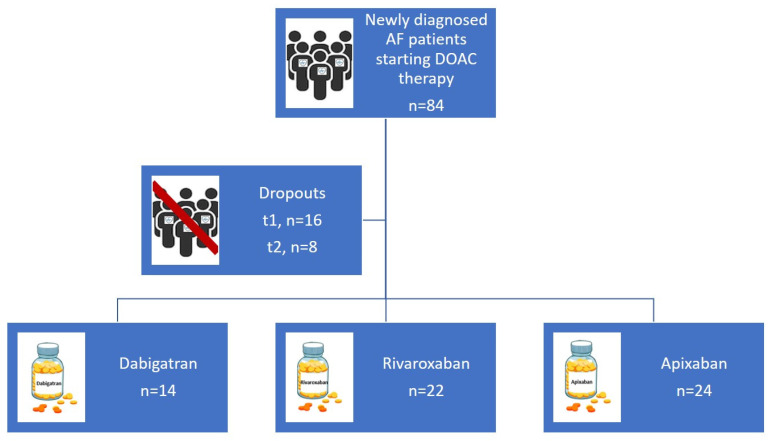
Analytic flowchart of patients enrolled so far in miR-CRAFT study. A total of 84 atrial fibrillation patients were initially enrolled. Twenty-four participants missed the follow-up visits (*n* = 16 for t1, *n* = 8 for t2). Among the 60 patients who completed the follow-up period, 14 are treated with dabigatran, 22 with rivaroxaban, and 24 with apixaban.

This is an ongoing study. So far, we have enrolled in our miR-CRAFT study 84 AF patients and 22 controls. Sixty patients have completed the 28-day follow-up period. An analytic flowchart of patient enrolment is presented in Figure 2. 

### 3.2. Population Characteristics

The main characteristics of study population are shown in Table 1. No major bleeding or thrombotic events were recorded. A total of 16 minor bleeding events occurred.

## 4. Discussion

This miR-CRAFT study investigates the dynamic expression of miRs and of DNA methylation during DOAC therapy and could guide precision medicine for DOACs. It employs an analysis of the expression of miRNome in AF patients treated with DOACs, the validation of the results of the total patient cohort, the identification of circulating miRs that reflect anticoagulation status, targeted DNA methylation analysis, and the integration, through bioinformatic analysis, of the regulatory network of miRNA-mRNA-genes for DOACs. 

Our results will deliver new knowledge regarding the pathways and mechanisms activated by DOACs and can reveal novel biological functions associated with DOAC pleiotropic effects. Specifically, the role of miRs has been most extensively studied in cancer. Therefore, the results of this miR-CRAFT study will potentially provide insights on onco-suppressive miRs that are potentially up-regulated by DOAC treatment, further supporting the anticancer effect of anticoagulants [30]. Similarly, we expect to identify the DOAC-induced miRs involved in the regulation of inflammatory pathways and characterize molecularly possible cascades promoting cardioprotection.

Despite the strengths of this miR-CRAFT study, several limitations should be acknowledged. The time-points for patient follow-up were selected to monitor DOAC-induced epigenetic changes on days 7 and 28, whereas long-term follow-up was not implemented in our study design. Currently, the optimal time-points for studying changes in time under the stimuli of epigenetic regulators are not specified. Thus, this project carries the risk of not identifying significant differences in miR expression or DNA methylation for the studied drugs. This risk is hindered by the novelty of the project, since no data exist on the association with DOAC-induced epigenetic changes. To minimize the risk, beyond the untargeted discovery phase, additional miRs will be analyzed in Phase II that will be carefully selected based on published results, where available, and on miR predictions from gene targets using NetworkAnalyst. The sample size was calculated to ensure the detection of a 1.5-fold change in miR expression with an 80% average power within groups; however, in sub-group analyses, the results should be interpreted with caution to avoid false positive results. MiRs expression and DNA methylation are dynamic states that can be affected not only by DOAC treatment but also by patient confounding factors, such as demographics, co-morbidities, or concomitant medications. To control for these covariates, in the Phase I untargeted analysis it will be included as possible matched cases and, for validation, cohort-adjusted statistical models will be used. 

DOACs have simplified thromboembolism prevention in AF, and there are indications that they exert effects beyond anticoagulation. Evidence shows that DOACs may also aid in atrial fibrosis and AF, providing overall qualities in AF therapy. It is thus a challenge to provide research-based evidence exploring the cross-talk of hypercoagulability and cardiac fibrosis. Notably, there is evidence that the activation of PAR signaling in the heart plays a pivotal role in cardiac inflammation, fibrotic response, and repair processes [31]. DOACs, by inhibiting factor Xa and thrombin, are recognized for preventing PAR signaling activation, thereby extending their effects beyond thromboembolism [2] []. Recent findings also propose that DOACs may deter atrial remodeling in rats through PAR signaling inhibition [32] and ameliorate cardiac fibrosis in AF mice [33]. The miR-CRAFT results can potentially be used towards knowledge in this direction.

## 5. Conclusions

DOACs have revolutionized oral anticoagulant therapy after the long-lasting dominance of vitamin K antagonists as the sole available oral drug class. Studying the epigenetic changes that accompany DOAC treatment holds promise in leading to the identification of DOAC-activated pathways, beyond anticoagulation. Our miR-CRAFT findings will be disseminated through scientific articles, as well as via attendance at national and international congresses or academic lectures/seminars/workshops. We expect that our miR-CRAFT design could be used to aid in unraveling mechanistic pathways for novel anticoagulant drugs that are currently undergoing clinical trials (factor XI inhibitors such as Milvexian, and Asundexian), as well as for other drugs that exert pleiotropic actions. 

## Figures and Tables

**Table 1:** Main characteristics of study cohort.

Demographic and Clinical Characteristics	AF Patients (*n* = 60)	Non-AF Controls (*n* = 22)
Gender (male, %)	35 (58.3)	11 (50.0)
Age, years (mean ± SD)	68.7 ± 11.8	57.9 ± 9.6
Hypertension (*n*, %)	39 (65.0)	10 (45.5)
Type 2 Diabetes (*n*, %)	17 (28.3)	4 (18.2)
Dyslipidemia (*n*, %)	31 (51.7)	11 (50.0)

## Data Availability

No new data were created or analyzed in this study. Data sharing is not applicable to this article.
